# A Debatable Poor-Law Plan

**Published:** 1920-04-10

**Authors:** 


### A Debatable Poor-Law Plan.

A question was raised in the House of Lords last week on the advocacy by the Ministry of Health of the gathering together of children from several Poor-Law Unions into one workhouse in the district. It is quite true that the present difficulty of building cottage homes is great, but the temporary plan advocated in place of this development is regarded with little favour by the State Children's Association, from which we have received a letter on the subject, which puts the case soberly, succinctly, and conclusively.

The Ministry of Health is rightly reinforcing the article of the Poor-Law Institutions Order, 1913 (suspended during the war), which requires the removal from workhouses of normal children over three years of age. In consequence, Boards of Guardians, who neglected to remove children from workhouses before the war, now find themselves faced with difficulty in providing for them, owing to the lack of existing accommodation and the high cost of building. It is therefore proposed, for instance, to collect the boys and girls from five West Cornish Unions, numbering 180 to 200, into the Truro Workhouse; those from six Wiltshire Unions and one Hampshire Union into Marlborough Workhouse; and those from five Devon and two Cornish Unions into the workhouse at Holsworthy.

### The Alternative.

The letter protests against such a travesty of the spirit of the Order, which sought to secure the removal of children from workhouse life and surroundings. "It is true that under the proposed scheme they would be free from the sometimes contaminating influences of adult workhouse inmates, but the dulness, monotony, and isolation of institutional life will be theirs even more markedly than when in the ordinary workhouse, from which at least they went out to the elementary schools, and sometimes to the Sunday Schools, Bands of Hope, etc., of the neighbourhood. Under this retrograde scheme the children will have to be taught within the workhouse building, in rooms never meant for educational purposes.

"A workhouse cannot be converted into a home by merely changing its name; large buildings and institutional discipline of work and play will not make up to a child for separation from family life and the interests born of affection.

"It is said that these would be only temporary arrangements, but experience proves that institutions, once established, take root and become difficult to break up.

"Many Guardians are much opposed to this suggestion and some boards have refused to take part in it.

"In spite of the difficulties which now confront them, it is still possible for each Board of Guardians to provide in individual ways for its own group of children, by better paid and extended boarding-out, by the emigration to Canada of suitable children through accredited agencies, by increased out-relief to widowed mothers to enable them to keep their children with them, by the establishment of small homes—a course still possible where Guardians will take the necessary trouble—and by the use of small homes already established in other Unions."

These methods will entail much more trouble than the collection of children into one workhouse, but no work of this character can be accomplished without pains.

